# Impact of specialist palliative care on utilization of healthcare and social services at the end-of-life: a nationwide register-based cohort study

**DOI:** 10.1093/eurpub/ckaf044

**Published:** 2025-05-28

**Authors:** Satu E Ahtiluoto, Timo P Carpén, Pirita T Forsius, Mikko S J Nuutinen, Nelli-Sofia A Nåhls, Pauliina M Kitti, Teija H Hammar, Harriet U Finne-Soveri, Tiina H Saarto

**Affiliations:** Palliative Care Center, Comprehensive Cancer Center, Helsinki University Hospital and University of Helsinki, Helsinki, Finland; Palliative Care Center, Comprehensive Cancer Center, Helsinki University Hospital and University of Helsinki, Helsinki, Finland; The Department of Healthcare and Social Welfare, Finnish Institute for Health and Welfare, Helsinki, Finland; Nordic Healthcare Group, Helsinki, Finland; Department of Oncology, Vaasa Central Hospital, The Wellbeing Services County of Ostrobothnia, Vaasa, Finland; Department of Oncology, Comprehensive Cancer Centre, Helsinki University Hospital and University of Helsinki, Helsinki, Finland; The Department of Healthcare and Social Welfare, Finnish Institute for Health and Welfare, Helsinki, Finland; The Department of Healthcare and Social Welfare, Finnish Institute for Health and Welfare, Helsinki, Finland; Palliative Care Center, Comprehensive Cancer Center, Helsinki University Hospital and University of Helsinki, Helsinki, Finland

## Abstract

Non-malignant diseases cause 60% of non-communicable diseases requiring palliative care, yet specialist palliative care services primarily focus on cancer. We investigated end-of-life healthcare and social services utilization among cancer and non-malignant patients, and, secondarily, access to specialist palliative care and its effect on services utilization. This retrospective, nationwide register-based study included all adults (*n* = 38 540) who died from non-communicable life-limiting diseases in Finland in 2019, categorized into neurodegenerative (31%), other non-malignant (36%), and cancer (33%) groups. Hospital was the most common place of death (61%). Healthcare utilization substantially increased during the final weeks of life in all groups but remained highest in cancer patients. Social services utilization was highest in neurodegenerative diseases. Specialist palliative care contact was significantly (*P* < .001) higher in cancer (30.1%) compared to neurodegenerative (10.9%) and other non-malignant (7%) diseases. Early (>30 days before death) compared to late/no specialist palliative care contact significantly reduced emergency care contacts (47.8% vs. 52.2%) and hospitalizations in secondary hospitals (24.7% vs. 33.7%), and increased specialist palliative care ward (15.5% vs. 1.5%) and hospital-at-home (36.8% vs. 3.4%) utilization during the final month (*P* < .001). Healthcare utilization was high in all disease groups, highest among cancer patients. Hospital was the most common place of death. Specialist palliative care contact was rare in non-malignant diseases. Early contact with specialist palliative care associated with lower emergency care utilization and secondary hospital inpatient care during the last month of life. These results highlight the necessity for timely equitable specialist palliative care services for all.

## Introduction

Global mortality reached 61 million people in 2023 [1], with the World Health Organization (WHO) estimating that annually over 56.8 million people worldwide require palliative care, with 45.3% requiring care during the last year of life and 54.7% prior to that [2]. The WHO estimates that non-communicable diseases cause approximately 73% of deaths and non-malignant diseases cause approximately 60% of the palliative care need in Europe [2]. In high-income countries leading causes of death include ischemic heart disease, Alzheimer’s disease and other dementias, stroke, cancers of lung, bronchus and trachea, and chronic obstructive pulmonary disease [3].

Palliative care focuses on relieving suffering and improving quality of life (QOL) of seriously ill patients and their families. The need is rapidly increasing due to population aging [2]. Historically, palliative services have mainly focused on cancer patients rather than patients with non-malignant diseases [4–6], leading to 32%–62% of cancer patients with metastatic disease receiving palliative care [7–9].

Among patients with advanced cancer, palliative care, especially early integrated palliative care improves QOL and reduces utilization of acute healthcare services at the end-of-life [10–15]. Cancer patients receiving early (>30 days before death) palliative care (mixed model generalist and specialist palliative care) were less frequently exposed to inappropriate end-of-life care including less emergency care and intensive care services compared to cancer patients receiving palliative care late (<30 days before death) or not at all [16].

Increasing evidence suggests potential benefits of specialist palliative care for patients with non-malignant diseases [17, 18]. A meta-analysis of 28 studies found palliative care interventions to decrease acute health care use and slightly decrease symptom burden [17]. A study comparing receipt and effect of palliative care for lung cancer and chronic obstructive pulmonary disease (COPD) patients reported that patients with lung cancer had more access to specialist palliative care during the last three months of life than did those with COPD but access to these correlated with fewer emergency room visits and fewer admissions to acute hospitals during the last month of life for both the groups [19].

Approximately 63 000 people died in Finland in 2023, mostly in older age due to life-limiting diseases [20]. The WHO estimates that in Europe 1337 patients per 100 000 habitants require palliative care, translating to about 75 000 people annually in Finland [2].

The WHO has stated that every country should have a national strategy and policies on palliative care development and implementation [2].

To our knowledge, no earlier studies exist that have utilized nationwide register-based data to investigate the end-of-life services utilization and place of death of decedents of all non-communicable causes of death expected to require palliative care, and how specialist palliative care services might affect other services utilization.

We aimed to investigate, on a nationwide level, the place of death, and healthcare and social services utilization at the end-of-life among patients with cancer and non-malignant diseases. The secondary aim was to study access to specialist palliative care and its association to healthcare and social services utilization at the end-of-life.

## Methods

### Study population

The study population was identified from the 2019 Causes of Death Register (Statistics Finland) [20] using the International Statistical Classification of Diseases and Related Health Problems 10th Revision (ICD-10) [21] diagnosis code stated as the primary cause of death for decedents aged ≥18 years. The ICD-10 codes chosen for this study included all non-communicable life-limiting diseases in which the need for palliative care is expected to arise during disease progression. After excluding 53 decedents who died outside Finland, the final study population consisted of 38 540 decedents. Patients were categorized into three groups based on the primary cause of death: neurodegenerative diseases, other non-malignant diseases, and cancers. The list of ICD-10 codes included are listed in Supplementary Table S1. The date and place (home, long-term care facility, hospital) of death were identified. The study was conducted and reported in accordance with the STROBE (Strengthening the Reporting of Observational Studies in Epidemiology) guidelines [22].

### Definition and utilization of healthcare and social services

Every individual living permanently in Finland has a personal identity code. The Finnish Healthcare Authority mandates all public and private healthcare and social service providers to register each service contact into the National Care Register using the individuals’ personal identity codes. Data recorded during healthcare and social services contacts, and by pharmacies (drug prescriptions and purchases) are stored securely in digital format in the Kanta Services Client data repository where they can be securely accessed by professionals and the individuals themselves. The registers provide information on socio-demographics, utilization of healthcare and social services, advance care directives, and medication prescriptions.

The definitions of healthcare and social services are presented in Supplementary Table S2. Data were collected from 1 January 2018 to 31 December 2019, on all clinic visits, contacts with and periods of inpatient care in primary, secondary, and tertiary healthcare and social services units, including long-term care facilities. In this study, data on secondary and tertiary healthcare units are combined into one variable, as are contacts with both primary and secondary emergency departments. Utilization of specialist palliative care services was identified by unit codes specific for each service provider.

All the data extracted from the registries were linked using the mandatory personal identification code for each patient, which were subsequently replaced with research numbers to pseudonymize the data.

To study the potential impact of early specialist palliative care contact on the utilization of healthcare and social services, the population was divided into two groups (1) early contact >30 days before death and (2) late contact ≤30 days prior to death or not at all.

### Healthcare services including specialist palliative care in Finland

Finnish residents are entitled to publicly provided healthcare, including primary, secondary, and tertiary healthcare, emergency services, and social welfare services. For the purposes of this study, we combined secondary and tertiary care data and refer to it collectively as “secondary care” throughout the article. At the time of the study, primary healthcare was provided by municipalities, secondary care by 20 secondary hospitals each serving their own hospital district area and tertiary care by five tertiary (university) hospitals each serving their own joint municipal catchment area (Supplementary Table S3).

Both general and specialist palliative care are publicly provided. Specialist palliative care is provided in specialized units by multidisciplinary teams led by doctors with specialist palliative care competence, and including registered nurses with palliative care training. Five tertiary hospitals and twelve secondary and primary hospitals provide specialist palliative care outpatient clinic services and consultation services to other medical care units. Municipalities provide specialist palliative clinic, inpatient and hospital-at-home care. Specialist palliative hospice care is provided by the third sector and three hospice units were active at the time of data collection. General palliative care is provided by some units in primary and secondary hospitals, primary healthcare, home care, and social service units but currently it is not possible to identify general palliative care in the registries.

### Determination of the palliative care decision

The ICD-10 code Z51.5 indicates the palliative care decision, referring to the situation where disease stabilizing or modifying medical treatments are limited to improve prognoses, and when focus is on comfort and palliative care. The first occurrence of the ICD-10 code Z51.5 was identified from the Care Register data and that date considered the date of the initial statement of the palliative care decision.

### Place of death and degree of urbanization

Place of death was determined from the 2019 Causes of Death Register data and categorized as home (private housing), long-term care facility or hospital (including specialist palliative care hospices, primary, secondary, and tertiary hospitals). Municipalities were categorized into urban, semi-urban, and rural as defined by Statistics Finland [20].

### Statistical analysis

Means, frequencies, and medians were used to examine differences between the three diagnosis groups in utilization of services. Pairwise Pearson's chi squared test was used to test for the differences in the categorical data between the groups. Mann–Whitney *U*-test was used to test for the differences between service use and the groups. *T*-test was used to compare mean age between different diagnosis groups. *P*-values of <.05 were considered statistically significant. All analyses were performed using Python (3.10—SDK v2) and its packages or IBM SPSS Statistics (version 29) software (SPSS Inc., Chicago, IL, United States).

## Results

### Characteristics of the study population

Basic characteristics are present in Table 1. The population consisted of 38 540 decedents, mean age at death was 80 years and 51% were female. Cause of death was neurodegenerative disease (31%), other non-malignant disease (36%), and cancer (33%).

**Table 1.** Basic characteristics of the people who died in Finland in 2019 due to diseases requiring palliative care

	All patients	Neurodegenerative diseases	Other non-malignant diseases	Cancers
Population, *n* (%)	38 540 (100)	11 902 (30.9)	13 759 (35.7)	12 879 (33.4)
Females, *n* (%)	19 704 (51.1)	7559 (63.5)	6179 (44.9)	5966 (46.3)
Mean age at death in years	80.4	86.4	80.6	74.8
Place of death, *n* (%)				
Home	5831 (15.1)	834 (7.0)	3575 (26.0)	1422 (11.0)
Long-term care facility	9244 (24.0)	5921 (49.7)	2444 (17.8)	879 (6.8)
Hospital	23465 (60.9)	5147 (43.2)	7740 (56.3)	10 578 (82.1)
Municipality type, *n* (%)				
Urban	24601 (63.8)	7725 (64.9)	8473 (61.6)	8403 (65.2)
Semi-urban	6749 (17.5)	2080 (17.5)	2374 (17.3)	2295 (17.8)
Rural	7144 (18.5)	2096 (17.6)	2873 (20.9)	2175 (16.9)
Z51.5 code^a^ stated, *n* (%)	8354 (21.7)	607 (5.1)	814 (5.9)	6933 (53.8)
Contact with specialist palliative care, *n* (%)	6130 (15.9)	1297 (10.9)	960 (7.0)	3873 (30.1)

Data are shown for the whole study population and separately for each three diagnosis group.

*n* = number of patients.

a ICD-10 code Z51.5 palliative care.

Hospital deaths were common in the total study population (61%) and in each diagnosis group. However, the diagnosis groups differed significantly (*P* < .001) in their distribution into the three place of death categories. Long-term care facilities were the most common place of death for neurodegenerative diseases decedents (50%) and hospital (82%) for cancer decedents (Table 1). Home as place of death was more common for decedents of other non-malignant diseases (26%) than for other disease groups.

### Utilization of healthcare services

**Figure 1.**
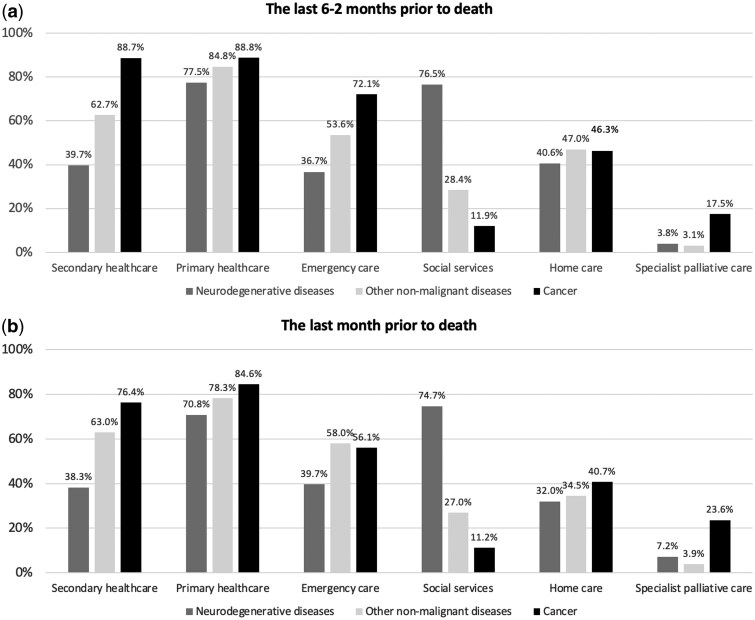
Proportions of patients in each diagnosis group who utilized the specified types of healthcare and social services during the (a) last six to two months and (b) last month prior to death in 2019.

In each diagnosis group, more than half of patients utilized primary and emergency care services during the last six to two months of life, with utilization predominating in cancer patients (Fig. 1a). During the final month of life emergency care utilization in patients with other non-malignant diseases increased and surpassed that of cancer patients (Fig. 1b). The use of secondary healthcare was significantly (*P* < .001) higher among patients with cancer compared with other disease groups during the last six to two months of life and significantly higher among patients with other non-malignant disease compared with patients with neurodegenerative disease (Fig. 1).

Hospitalizations and the utilization of emergency care increased remarkably in patients with other non-malignant disease and cancer during the final weeks of life (Supplementary Fig. S1).

Cancer patients had the highest median inpatient days in secondary hospitals and on specialist palliative care wards during the last six months of life (Table 2).

**Table 2.** Utilization of healthcare and social services during the last six months and last one month prior to death of those who died in Finland in 2019 due to diseases requiring palliative care

	All patients	Neurodegenerative diseases	Other non-malignant diseases	Cancers
Secondary hospital inpatient days, median (IQR)				
The last 6 months	8.0 (13)	5.0 (8)	8.0 (14)	10.0 (13)
The last month	6.0 (8)	4.0 (6)	6.0 (8.8)	6.0 (9)
Primary hospital inpatient days, median (IQR)				
The last 6 months	17.0 (30)	15.0 (33)	18.0 (32)	17.0 (28)
The last month	12.0 (19)	11.0 (20)	11.0 (17)	13.0 (19)
Specialist palliative care ward inpatient days, median (IQR)				
The last 6 months	12.0 (21.2)	10.0 (19)	9.0 (18)	13.0 (22)
The last month	11.0 (17)	9.0 (18)	9.0 (17)	12.0 (17)
Social service inpatient days, median (IQR)				
The last 6 months	179.0 (89)	180.0 (14)	166.5 (139)	79.0 (156)
The last month	30.0 (2)	30.0 (0)	30.0 (7)	30.0 (13)

The median length of inpatient care in days is shown for the whole population and for each diagnosis group separately.

The median inpatient days are calculated only for patients with ward stays during the specified period.

IQR = interquartile range.

### Utilization of social services

Utilization of social services was significantly (*P* < .001) higher, and the median length of social care services was significantly (*P* < .001) longer in the neurodegenerative diseases group during the last six to two months and during the last month prior to death than in the other groups (Fig. 1 and Table 2).

### Specialist palliative care utilization and its impact on the utilization of healthcare and social services

Specialist palliative care utilization was low overall with only 6130 (16%) of the total population receiving this type of service. A significantly (*P* < .001) higher proportion of cancer (30.1%) patients received specialist palliative care compared to patients with neurodegenerative (10.9%) or other non-malignant diseases (7%). The difference between neurodegenerative and other non-malignant diseases groups was smaller, but statistically significant (*P* < .001) in favour of neurodegenerative diseases (Table 1).

The median time from the first specialist palliative care contact to death was 72 days (IQR 198). Early contact (>30 days prior to death) occurred in 4088 (11%) and late contact (≤30 days/no contact) in 34 452 (89%) of patients. The median times from the first specialist palliative care contact to death were 152 days (IQR 238) and 9 days (IQR 15) for patients with early contact and late contact, respectively.

Specialist palliative care use increased steadily among cancer patients during the last months of life, with a notable rise during the last week of life compared to the prior week (17% vs. 10%). A similar but less pronounced increase was observed for patients with neurodegenerative (6% vs. 2%) and other non-malignant diseases (3% vs. 1%) during the last week compared to the prior week (Supplementary Fig. S1).

The median of inpatient days in specialist palliative care was highest for cancer patients, compared to the other diagnosis groups during the last six to two months and the final month of life (Table 2).

**Figure 2.**
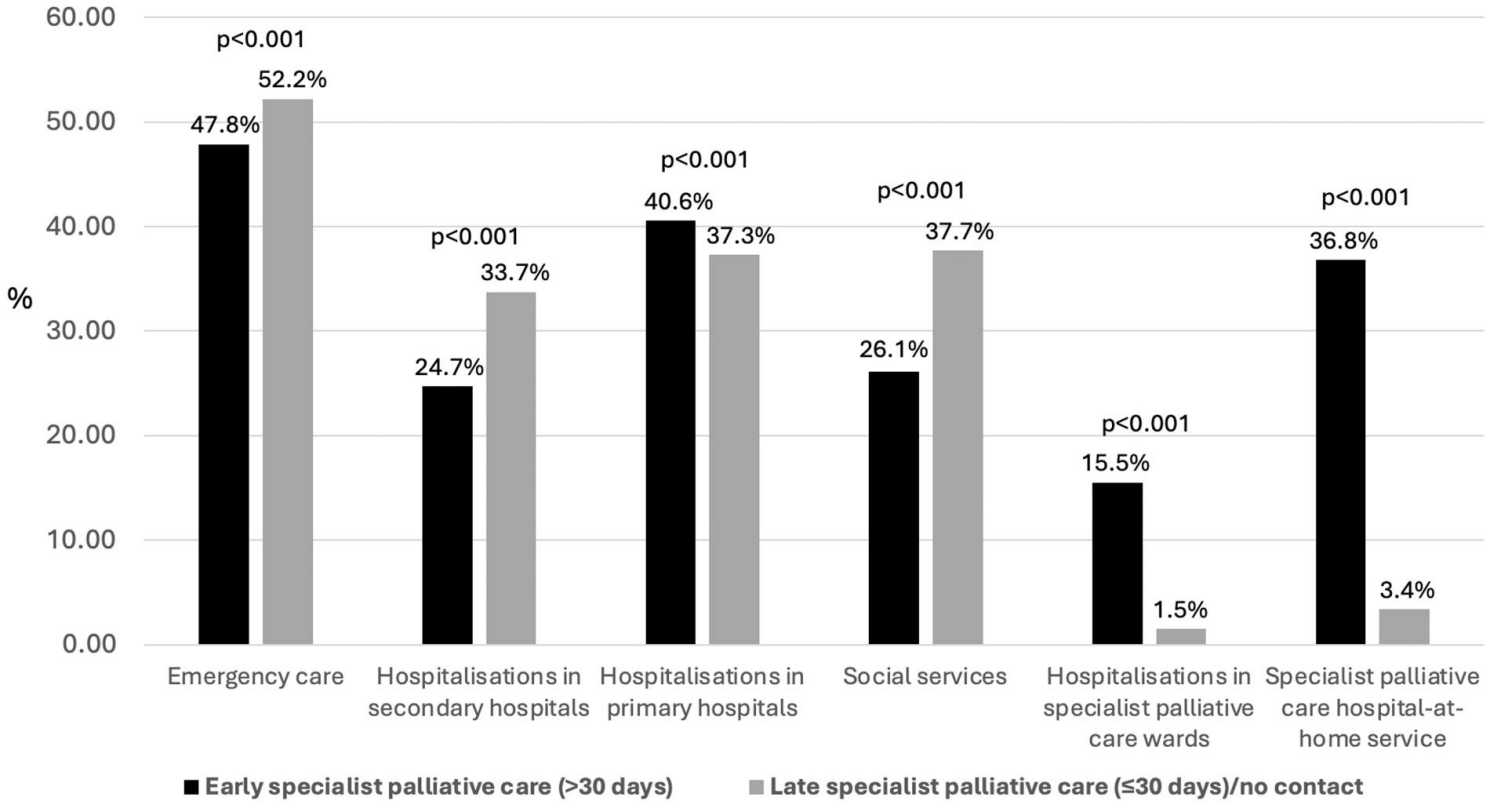
The impact of the timing of the first specialist palliative care contact on the utilization of health care and social services during the last month prior to death in 2019.

Early specialist palliative care contact compared to late/no contact was associated with significantly fewer emergency care contacts, hospitalizations in secondary hospitals, and social services contacts, and significantly more primary care hospital, specialist palliative care ward, and hospital-at-home utilization during the final month of life (Fig. 2).

## Discussion

This nationwide study provides valuable insights into end-of-life healthcare and social service utilization patterns and the place of death of the Finnish adult population who died from life-limiting diseases in 2019. Primary causes of death were quite evenly distributed into three disease groups: neurodegenerative diseases, other non-malignant diseases, and cancers. Healthcare services utilization was high in all groups and increased during the final weeks of life. Hospital was the most common place of death, while dying at home was rare. Early access to specialist palliative care services seemed to reduce utilization of acute hospital services. However, access to specialist palliative care services was low overall and mainly concerned cancer patients.

In the present study, emergency care utilization and hospitalizations in both secondary and primary hospitals escalated during the last weeks of life in all diagnosis groups resulting in a high hospital death rate. In this Finnish cohort, hospital remained the most common place of death, accounting for nearly two-thirds of patients overall and an even higher proportion among cancer patients. Patients with neurodegenerative diseases died more often in long-term care facilities (50%), reflecting the dependency developed in cognitive disorders. Overall, dying at home was rare. Even though approximately a quarter of patients with other non-malignant diseases died at home, taking into consideration the low level of specialist palliative care contacts in this group, this potentially reflects sudden deaths without specialist palliative care support. Internationally, there are significant country-level variations in place of death for patients who died of diseases indicative of palliative care need, with hospitals being the predominant place of death in some nations like France, Spain, South Korea, and Hungary, while home and long-term care facilities were more common in Mexico and the Netherlands [23]. Finland's high hospital death rates may relate to insufficient end-of-life care resources and expertise, as well as geographical challenges, including a relatively large area with low population density, in providing specialized home-based palliative care services nationwide. In addition to this, 40% of households in Finland are single-person households decreasing the possibility for a carer from the same household to help the patient.

In our study population, only every sixth patient had contact with specialist palliative care services. Cancer patients utilized specialist palliative care significantly more throughout the last six months of life than the other groups. According to previous literature, palliative care utilization of patients with metastatic cancer has varied between 32% and 62% as compared to 30% in the present cohort [7–9]. In our previous publication, 37% of cancer patients who were treated at university hospital cancer centres had specialist palliative care contact [24]. The slightly lower proportion in the present cohort may be, at least partly, due to the fact that our cohort represented an unselected nationwide sample of deceased patients.

The low palliative care utilization in non-cancer patients is consistent with previous studies demonstrating the historical focus of palliative care on cancer patients, despite the substantial need for such services among those with neurodegenerative and other non-malignant conditions [5, 25, 26]. The WHO estimates that a large proportion of those in need of palliative care worldwide are those with non-malignant diseases, highlighting the significant unmet need in these populations [2]. The results of this study support earlier findings that there are major challenges in recognizing approaching death and in providing timely palliative care, especially for non-cancer populations [4, 5, 26].

In our cohort, patients with an early (>30 days before death) specialist palliative care contact had significantly fewer emergency care contacts and hospitalizations in secondary hospitals during the last month of life compared to those with late or no specialist palliative care contact. Early specialist palliative care contact was associated with more hospitalizations in specialist palliative care wards and increased contacts with specialist palliative care hospital-at-home services. These findings suggest that early specialist palliative care involvement may have facilitated more planned and coordinated end-of-life care, shifting healthcare service utilization away from acute, unplanned secondary hospital care toward care in specialist palliative care units.

Even though we defined the cutoff for early palliative care at more than 30 days before death, in our cohort the mean time from the first specialist palliative care contact to death was 72 days for the total population and 152 days for patients in the early palliative care group. Thus, our findings support the early integration of palliative care in the care of patients with life-limiting illness. Likewise, Hui *et al.* have demonstrated that patients referred to palliative care more than three months before death experienced fewer emergency department visits and hospital admissions in their last 30 days, and fewer in-hospital deaths [27].

A similar pattern has been observed in various healthcare systems, with studies suggesting that a significant proportion of acute care visits and hospitalizations could potentially be avoided through the provision of comprehensive palliative care services [17, 28]. A large cohort study by Obermeyer *et al.* on cancer patients from United States found that hospice care substantially decreased hospitalizations, intensive care admissions, and invasive procedures in the final stages of life [29]. A systematic review and meta-analysis by Kavalieratos *et al.* revealed that palliative care interventions can lower healthcare utilization among patients with life-limiting illnesses [30]. In addition, a meta-analysis of 28 trials including patients with primarily non-malignant diseases demonstrated less emergency department use and hospitalizations after receipt of palliative care interventions [17].

The palliative care diagnostic code (Z51.5) was only documented for a minority of patients in our total cohort, but for more than half of cancer patients. This likely underestimates the true palliative care decision making, as the code was not yet systematically used, especially outside oncology. Prior research, however, suggests that early palliative care decision making can increase referral to specialist palliative care services and reduce acute care utilization near the end-of-life, highlighting the importance of timely palliative care recognition across all advanced, progressive diseases [15, 24].

The findings of this study emphasize the need to formulate standardized referral criteria to palliative care in all life-limiting diseases, both non-malignant and malignant diseases. Additionally, it seems beneficial to ensure that palliative care is integrated into standard care early in the disease course of these diseases. Policy changes are acutely needed to ensure these changes in clinical practice are realized and the provision of equitable access to specialist palliative care services for all patients suffering from life-limiting diseases is accomplished.

Strengths of this study include a large nationwide cohort including all deceased patients with causes of death expected to result in the need for palliative care. To our knowledge, such nationwide cohorts are scarce. The study cohort was formed from the national standardized, high-quality death register. Data on utilization of healthcare and social services were obtained from mandatory, comprehensive healthcare registries. Specific unit codes enabled us to identify utilization of specialist palliative care services. As limitations, the data are retrospective, the register data lacks information on multimorbidity, and the specific data on healthcare interventions.

## Conclusions

This comprehensive nationwide study provides insight into the end-of-life trajectories of patients with life-limiting diseases. The results indicate that utilization of healthcare services at the end-of-life was high in most patients with life-limiting diseases and increased with approaching death. Early specialist palliative care contact seems to reduce the use of emergency and secondary healthcare services and increase access to end-of-life care in specialist palliative care services. However, specialist palliative care services are not equally available for all patients, as cancer patients have better access. The results of this nationwide study underline the need for further development of high-quality specialist palliative care services to provide timely and equitable specialist palliative care for all patients suffering from life-limiting diseases.

## Supplementary Material

ckaf044_Supplementary_Data

## Data Availability

The data that support the findings of this study are available from the Finnish Institute for Health and Welfare, but restrictions apply to the availability of these data, which were used under license for the current study, and so are not publicly available. Data permits can be requested from the Finnish Social and Health Data Permit Authority, Findata (info@findata.fi).
