# Supplementary material for: Impact of specialist palliative care on utilization of healthcare and social services at the end-of-life: a nationwide register-based cohort study
Source: Eur J Public Health. 2025 May 28;35(5):828–34. doi: 10.1093/eurpub/ckaf044 (PMC12529283; doi:10.1093/eurpub/ckaf044)
Supplement: ckaf044_Supplementary_Data [file ckaf044_supplementary_data.zip › ckaf044_Supplementary_Data/ejph-2024-12-om-0939-File006.docx]

SUPPLEMENTARY MATERIAL

| **Table S1. Diagnosis groups according to underlying cause of death by ICD-10 classification.** | |
| --- | --- |
| Diagnosis group | ICD-10 codes |
| Cancers | C00-C97 |
| Neurodegenerative diseases | Cognitive disorders (F00, F01, F03, G30, R54)  Central nervous system diseases (G10-13, G20, G23, G30-32, G35-37, G70-73) |
| Non-malignant diseases | Diabetes (E10-E14)  Hypertension induced renal and heart diseases (I11-14)  Pulmonary heart disease (I27)  Cardiomyopathy (I42)  Heart failure (I50)  Sequelae of cerebrovascular disease (I69)  Chronic lung diseases (J43, J44, J84, J96.1, J96.9)  Liver diseases (K70.2, K70.3, K72.1, K72.9, K74)  Kidney diseases (N18, N19) |

| **Table S2. The utilization of health care and social services were obtained from the Care Register for Health Care, Register of Primary Health Care visits and Register of Social assistance*. The services were categorized into to the secondary health care, primary health care, emergency care, social services, home care, hospital at home and specialist palliative care.** | | | |
| --- | --- | --- | --- |
| Variable | Care Register for Health Care | Register of Primary Health Care visits | Register of Social assistance |
| Secondary/tertiary health care | EA ≠ 98 OR  (EA = 98 AND (PALA ≠ 1 AND YHTEYSTAPA ≠ R80)) |  |  |
| Primary health care | EA = 98 AND (PALA = 1 OR YHTEYSTAPA = R80) | KAYNTI_PALVELUMUOTO ≠ T40, T41, T42, T43 |  |
| Emergency care | PALA = 91 OR  ((KIIREELLISYYS = 5, 6) AND (YHTEYSTAPA = R10, R20, R30, R41, R52, R56 OR R90)) | KAYNTI_KIIREELLISYYS = 1 |  |
| Social services |  |  | PALVELUALA ≠ 7 |
| Home care | PALA = 7 | KAYNTI_PALVELUMUOTO = T40, T41, T42 | PALVELUALA = 7 |
| Hospital-at-home | Contact linked to specialist palliative care unit (hospital-at-home unit) | Contact linked to specialist palliative care unit (hospital-at-home unit) | Contact linked to specialist palliative care unit (hospital-at-home unit) |
| Specialist palliative care | Contact linked to specialist palliative care unit | Contact linked to specialist palliative care unit | Contact linked to specialist palliative care unit |
| EA = Erikoisala (Speciality); YHTEYSTAPA = Yhteystapa (Contact type); PAL= Palveluala (Service branch); KAYNTI_PALVELUMUOTO = Palvelumuoto (Service type); KAYNTI_KIIREELLISYYS = Kiireellisyys (Urgency of care); PALVELUALA = Palveluala (Service branch); KIIREELLISYYS = Kiireellisyys (Urgency of care). Data of one of the specialist palliative care wards (Villa Apila) was gathered from the Register of Social assistance, because in 2019 the ward (Villa Apila) was technically under the Register of Social Assistance, instead of the Care Register of Health Care.  * Register descriptions, The Finnish Institute for Health and Welfare (THL), <https://thl.fi/en/web/thlfi-en/statistics-and-data/data-and-services/register-descriptions> | | | |

| **Table S3. Hospital districts and university hospitals.** | |
| --- | --- |
| University hospital | Hospital district |
| Helsinki University Central Hospital | Helsinki and Uusimaa Hospital District |
|  | South Karelia Hospital District |
|  | Kymenlaakso Hospital District |
|  | Päijät-Häme Hospital District |
| Tampere University Hospital | Pirkanmaa Hospital District |
|  | South Ostrobothnia Hospital District |
|  | Kanta-Häme Hospital District |
| Oulu University Hospital | North Ostrobothnia Hospital District |
|  | Kainuu Hospital District |
|  | Central Ostrobothnia Hospital District |
|  | Lappi Hospital District |
|  | Länsi-Pohja Hospital District |
| Kuopio University Hospital | Pohjois-Savo Hospital District |
|  | Etelä-Savo Hospital District |
|  | Itä-Savo Hospital District |
|  | Central Finland Hospital District |
|  | North Karelia Hospital District |
| Turku University Hospital | Satakunta Hospital District |
|  | Vaasa Hospital District |
|  | Varsinais-Suomi Hospital District |
