# Supplementary figures and images for: Impact of specialist palliative care on utilization of healthcare and social services at the end-of-life: a nationwide register-based cohort study
Source: Eur J Public Health. 2025 May 28;35(5):828–34. doi: 10.1093/eurpub/ckaf044 (PMC12529283; doi:10.1093/eurpub/ckaf044)

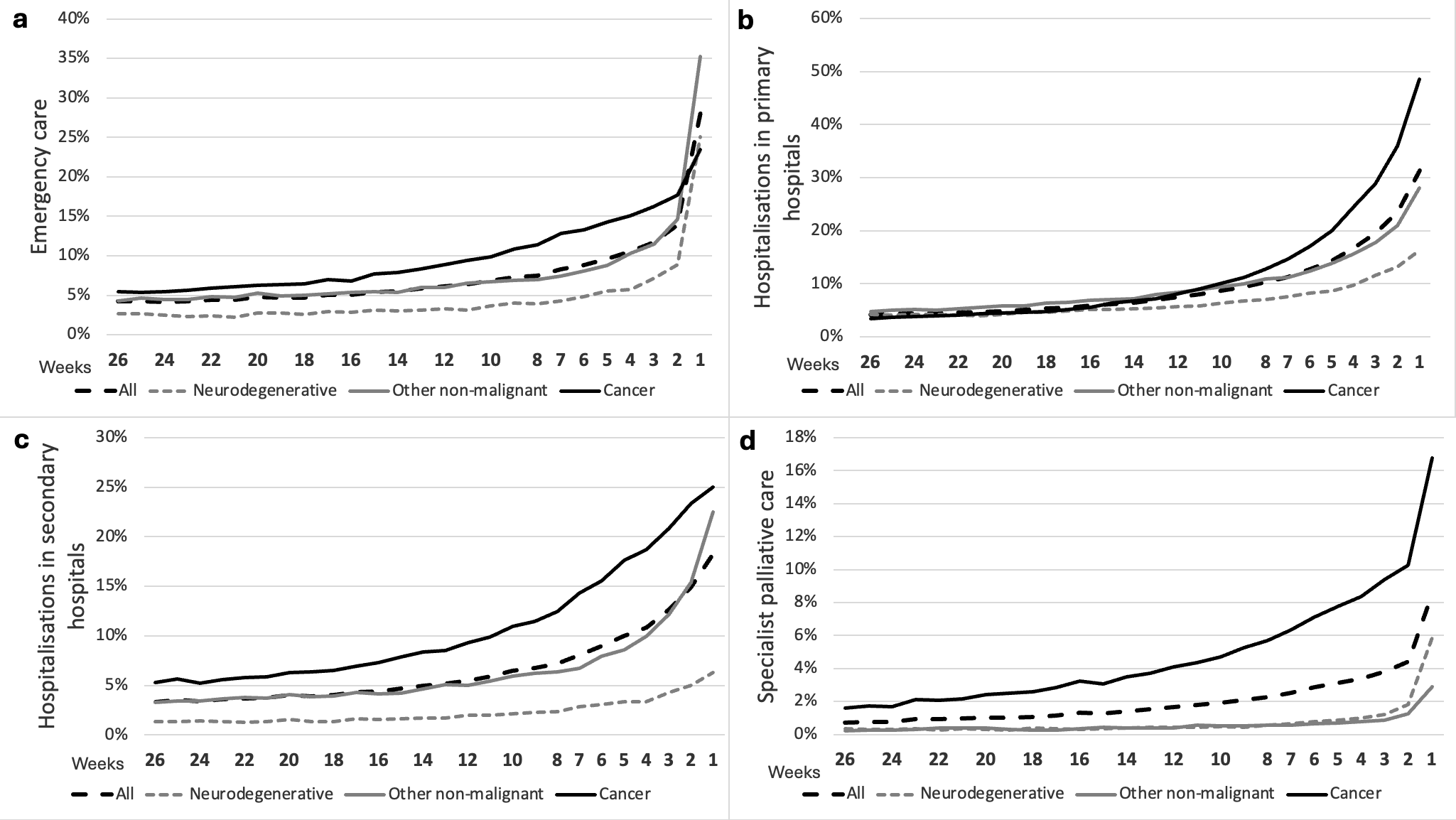

Supplement: ckaf044_Supplementary_Data [file ckaf044_supplementary_data.zip › ckaf044_Supplementary_Data/ejph-2024-12-om-0939-File007 (3).tiff]
